# A cost study for mobile phone health surveys using interactive voice response for assessing risk factors of noncommunicable diseases

**DOI:** 10.1186/s12963-021-00258-z

**Published:** 2021-06-28

**Authors:** Andres I. Vecino-Ortiz, Madhuram Nagarajan, Kenneth Roger Katumba, Shamima Akhter, Raymond Tweheyo, Dustin G. Gibson, Joseph Ali, Elizeus Rutebemberwa, Iqbal Ansary Khan, Alain Labrique, George W. Pariyo

**Affiliations:** 1 Department of International Health, Johns Hopkins Bloomberg School of Public Health, 615 N. Wolf Street., Suite E8620, Baltimore, MD USA; 2 Makerere University School of Public Health, Kampala, Uganda; 3 Institute of Epidemiology, Disease control and Research, Dhaka, Bangladesh

**Keywords:** Mobile phone surveys, Noncommunicable chronic diseases, Cost study, Surveillance

## Abstract

**Background:**

This is the first study to examine the costs of conducting a mobile phone survey (MPS) through interactive voice response (IVR) to collect information on risk factors for noncommunicable diseases (NCD) in three low- and middle-income countries (LMIC); Bangladesh, Colombia, and Uganda.

**Methods:**

This is a micro-costing study conducted from the perspective of the payer/funder with a 1-year horizon. The study evaluates the fixed costs and variable costs of implementing one nationally representative MPS for NCD risk factors of the adult population. In this costing study, we estimated the sample size of calls required to achieve a population-representative survey and associated incentives. Cost inputs were obtained from direct economic costs incurred by a central study team, from country-specific collaborators, and from platform developers who participated in the deployment of these MPS during 2017. Costs were reported in US dollars (USD). A sensitivity analysis was conducted assessing different scenarios of pricing and incentive strategies. Also, costs were calculated for a survey deployed targeting only adults younger than 45 years.

**Results:**

We estimated the fixed costs ranging between $47,000 USD and $74,000 USD. Variable costs were found to be between $32,000 USD and $129,000 USD per nationally representative survey. The main cost driver was the number of calls required to meet the sample size, and its variability largely depends on the extent of mobile phone coverage and access in the country. Therefore, a larger number of calls were estimated to survey specific harder-to-reach sub-populations.

**Conclusion:**

Mobile phone surveys have the potential to be a relatively less expensive and timely method of collecting survey information than face-to-face surveys, allowing decision-makers to deploy survey-based monitoring or evaluation programs more frequently than it would be possible having only face-to-face contact. The main driver of variable costs is survey time, and most of the variability across countries is attributable to the sampling differences associated to reaching out to population subgroups with low mobile phone ownership or access.

**Supplementary Information:**

The online version contains supplementary material available at 10.1186/s12963-021-00258-z.

## Contributions to the literature

- This is the first study describing the costs of using national-representative mobile phone surveys to monitor chronic conditions in three lower- and middle-income countries.
- This study identifies the main driver of variable costs for the deployment of these surveys.
- This study identifies populations that are hard to reach and therefore, lead to an increase in the costs of deploying these surveys, providing alternatives to collect data from these hard-to-reach populations.

## Background

Noncommunicable diseases (NCDs) are increasing in low- and middle-income countries (LMIC) due to the epidemiologic and nutritional transition, and the change in the population pyramid, phenomena already experienced in high-income nations [1, 2]. Governments, international agencies, and research organizations are increasingly discussing NCDs as the next challenge on the global health horizon. Noncommunicable diseases currently account for two thirds of all deaths taking place in LMICs [3, 4], but only receive 1% of global health funding [4].

In order to address the increasing burden of NCDs in LMICs, robust data streams are needed to prioritize policy options [5]. Countries need strong monitoring and evaluation systems to periodically assess risk factors. In LMICs, the high costs and logistic challenges associated with conducting household surveys, a common monitoring approach, may result in delays in data collection, or even in the omission of such surveys altogether [6].

Population health surveys administered through mobile phone technology (mobile phone surveys, or MPS) are a relatively new tool which can potentially obtain data on NCD risk factors in a timely manner [7]. If these surveys can be shown to collect robust risk factor data at a lower cost than existing population-based approaches, they could favorably shift the calculus of return on investment (ROI) of deploying these types of surveys in LMICs [8, 9]. While MPS cannot currently replace face-to-face household surveys due to constraints around collection of anthropometric or biochemical data, they have the potential to rapidly reach a large number of people in a dispersed set of locations. The shift in ROI and the possibility of near-real-time updates allows for more frequent deployments of MPS or even for continual deployment for a core set of survey questions and indicators. The United Nations has estimated that the use of MPS for NCD monitoring in LMICs may result in a reduction of up to 60% in survey costs [10].

Earlier studies have explored the sampling requirements, implementation, and effectiveness of NCD risk factor surveillance when conducted through surveys employing interactive voice response (IVR), consisting of prerecorded audio messages [11–18]. Some of these studies have shown that population surveys conducted through mobile phones can be less expensive than face-to-face surveys [19].

The Johns Hopkins University mHealth Initiative and the Bloomberg Philanthropies Data for Health Initiative (D4H) [20], along with local collaborators in Bangladesh, Colombia, and Uganda, have assessed the feasibility and validity of NCD risk-factor data collection through MPS using IVR in these countries through placing calls to randomly generated mobile phone numbers [21]. Nested in these surveys, we assessed the potential costs of developing and deploying one such MPS over a period of 1 year, using information about costs and resource utilization captured during our previous surveys. The aim of this study was to provide decision makers and funders information on the accounting average costs of conducting MPS for NCD risk factors in LMICs, to assess the economic feasibility and the related potential financial implications of its implementation.

## Methods

We conducted a microcosting analysis of the economic costs incurred if population-representative IVR surveys were implemented at the national level in three countries: Bangladesh, Colombia, and Uganda. Microcosting analyses have previously been used to appraise the costs of conducting population surveys [19] and other types of mHealth interventions [22].

This microcosting study was done from the perspective of the payer/funder, with an analytic horizon of 1 year, where an initial phase of adaptation and validation of the survey questions and its structure was conducted along with one deployment of the full IVR survey at the national level. For this reason, no discounting was used in this study.

For this microcosting study, we used direct resource utilization inputs for (1) the required resources to adapt and validate a full IVR survey and its deployment and (2) the number of required survey calls that would need to be deployed in order to have a nationally representative survey of the adult population for each of the three countries studied. Regarding costs, we calculated (1) fixed costs, which are costs incurred to set up and conduct field adaptation and validation of IVR surveys (these represent the work required to conduct the formative and technology testing phase for these surveys), and (2) variable costs, which are incurred in the deployment of the IVR surveys (including associated incentives) [23].

This study has been approved by the Institutional Review Board of the Johns Hopkins School of Public Health, the Ethics Committee of the Institute of Public Health of Universidad Javeriana in Colombia, the Institutional Review Board of the Institute of Epidemiology, Disease Control and Research in Bangladesh, and the Institutional Review Board of Makerere University School of Public Health in Uganda.

### Sampling

To calculate the sample size estimates for the projected nationally representative IVR surveys, we first used the Stepwise Approach to Surveillance (STEPs) surveys sample size calculator provided by the World Health Organization [24] to calculate the overall sample size for nationally representative STEPS surveys in each of the three countries, assuming a 95% confidence level, 0.05 margin of error, and 50% of estimated prevalence of risk factors. Moreover, we assessed the specific sample sizes required for each of eight age-sex categories sampled in STEPs surveys (consisting of both males and females, stratified in four age categories: 18–29, 30–44, 45–59, and 60 or older) [25].

Parameters on average time for both complete and incomplete surveys as well as paid calls required per complete survey were obtained from the IVR MPS study (Table 1). Data for this study was collected in Bangladesh between July 6, 2018, and February 18, 2019; in Colombia between November 14, 2018, and January 25, 2019; and in Uganda between June 22, 2018, and January 9, 2019.

**Table 1:** Calls sample, calls needed per completed survey by country

Country	Sample size		Price per minute of call (USD)	Average minutes of an incomplete call	Average minutes of a complete call	Paid calls needed per completed survey
	Total sample of calls	Subsample of young adults (< 45 years)				
Bangladesh	20,467	8531	0.05	1	11	41
Colombia	5800	3410	0.05	2	10	46
Uganda	4947	3701	0.10	1	10	15

Source: Total sample of calls is the number of calls estimated to obtain a sample of calls that is representative of the age-sex population distribution. Subsample of calls is the number of calls estimated to obtain a sample of calls that is representative of the population that is less than 45 years old. Calls needed per completed survey represent the number of paid calls required to obtain in average one complete survey

Using these parameters, we calculated weights based on the differences between the age-sex population distribution of the completed IVR surveys in each country and their corresponding national age-sex population distribution [23]. Weights were needed because some age-sex groups have lower levels of mobile phone use (low response rate). A larger number of calls are therefore necessary to achieve the minimum sample for some demographic strata.

Unlike in face-to-face surveys, the deployment of IVR surveys used random digit dialing or RDD [26]. RDD creates random digits that comprise a random phone number aiming at a phone number selection with less risk of being biased. Since we did not use quota-sampling, if a given age-sex category has already reached its target sample size, it will continue accepting surveys for that age-sex group until both of the following happen: (1) the total sample size (adding up for all age-sex categories) is achieved and (2) the specific sample size required for all other age-sex categories is achieved [26]. For example, this implies that survey among younger male respondents, who have higher rates of mobile phone use, is likely to yield a greater proportion of surveys completed than their corresponding proportion in the population. Conversely, age-sex categories with less mobile phone use (older women, for example) are less likely to be sampled and consequently require more outgoing calls and a greater number of completed surveys in order to achieve the target sample sizes. This sampling issue results in higher costs—the cost of the incentives provided for additional completed surveys, in addition to the costs of extra calls being made and extra survey-time—to reach desired sample size for all age-sex strata.

As this study was conducted under a scenario of non-quota sampling, quota sampling might reduce further the costs of deploying these surveys.

### Resource utilization

The MPS project that served as a basis for this costing analysis was divided into two phases: formative work and technology testing. Briefly, the formative work consisted of focus group discussions and user groups to validate translation of questionnaire, to ensure it is comprehensible, and to adapt the questionnaire by using country-specific examples. Using the adapted questionnaire and IVR platform from the formative work, the technology testing phase sent out the IVR surveys to RDD participants to confirm that the platform could successfully send surveys and incentives and that the data was extractable from the platform.

The calculation of resource utilization in this study is drawn from the actual resources used during these two phases. Resource utilization associated to one-time costs during the formative phase represents the actual resource utilization the local partners experienced. Resource utilization associated with the survey deployment (i.e., variable costs including calls deployed, and incentives paid) was estimated based on the sampling strategy described above and the response rates found during the project.

### Cost inputs

Costs inputs for the initial formative and adaptation work were based on actual costs incurred by in-country partner institutions and from the IVR technology platform providers (setup costs). Fixed costs related to platform programing and setup and the previous formative work were obtained in local currency units (LCU) from our collaborators and translated to 2017 United States dollars (USD).

Variable costs, which were charged by survey providers and consist of survey-time and incentives, are presented only in USD because survey providers generate charges in this currency. Since there are no local market forces involved (countries would pay for MPS in USD regardless of their purchasing power parity), we decided to present variable costs only in USD$ (Table 1).

Table 1 shows the inputs used in our estimation, including call sample sizes, calls needed per completed survey by country, average minutes for an incomplete call, average minutes for a complete call, and price per minute. Further data on age-sex distribution by country for complete calls, and calls in which respondent answered the age question (both complete and incomplete) with fixed incentive are provided in Appendix 1.

### One-way sensitivity analyses and subgroup analysis

One-way sensitivity analyses were performed on the primary factors affecting variable costs, namely survey pricing structure, and type of incentive offered. In the former, we assessed two scenarios: (1) price per minute and (2) a flat fee per completed survey. For the first scenario, we calculated the average number of calls needed per completed survey (that is the number of missed calls and incomplete surveys to have one complete survey) and the average duration of both complete and incomplete surveys. In a price per minute scenario, the payer/funder pays for both the completed and incomplete calls on an as-is basis. In the second scenario, we calculated the costs when the provider charges a flat fee per completed survey, thus internalizing the costs of incomplete surveys from the payer/funder.

When reporting the costs of incentives offered, we assessed two different scenarios: (1) incentive per completed survey and (2) lottery incentive. Incentives were provided in all cases as mobile phone airtime credits. For the first incentive scenario, we used the amount and type of incentive provided per completed survey as cost inputs, i.e., 0.6 USD, 1.58 USD, and 1.34 USD per completed survey for Bangladesh, Colombia, and Uganda, respectively, and the estimated number of complete surveys required from the sampling obtained above. In the second scenario, the incentive for participants consisted of the chance of “winning” a lottery for mobile phone credit. One of every 20 completed surveys would receive the equivalent of 6 USD, 15.8 USD, and 26.8 USD for Bangladesh, Colombia, and Uganda, respectively [26]. This corresponds to 10X the incentive per completed survey in the case of Bangladesh and Colombia and 20X the incentive per completed survey in the case of Uganda. The amounts in each country were decided in consultation with local experts and to align with incentives used in other studies in the country. As these were the actual structures and amounts of the incentives offered during the formative phase, in this study, we have used the actual response rates obtained during the trials.

Given that costs increased significantly when trying to capture older populations (which have lower mobile phone ownership), we also assessed and compared variable costs for a population-level, nationally representative survey of all adults versus its counterpart only for adults aged less than 45 years old. This manuscript adheres to the Consolidated Health Economic Evaluation Reporting Standards (CHEERS) [27].

## Results

**Fig. 1:**
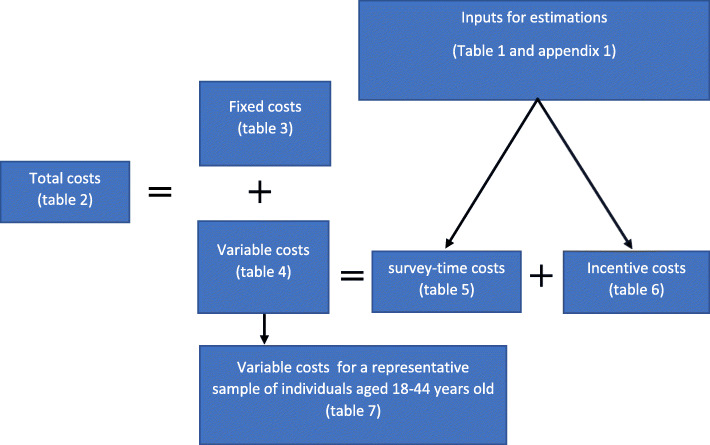
Cost components assessed in this study. The different cost components estimated to guide the reading of the paper as it relates each of the components to the respective tables

We calculated the target sample of calls required for Bangladesh, Colombia, and Uganda to be 20,467, 5800, and 4947 completed IVR surveys, respectively. In Fig. 1, we present the different cost components assessed and how they are presented in the tables included in this study.

In Tables 2 and 3, we present the estimated total and fixed costs respectively across the three countries—Bangladesh, Colombia, and Uganda—in USD. From these numbers, we see that Bangladesh has the highest total and fixed costs ($203,122 and $74,455, respectively), followed by both Uganda ($84,282 and $53,976, respectively) and Colombia ($86,049 and $47,038, respectively).

**Table 2:** Distribution of total costs across countries

Country	Total costs in US dollars
Bangladesh	203,122
Colombia	84,282
Uganda	86,049

Fixed costs were primarily incurred in local currency unit (LCU). Hence our reporting converts from LCU into 2017 US Dollars

^#^Using variable costs under scenario of price per completed survey and fixed incentive per survey

**Table 3:** Distribution of fixed costs across countries

Country	Fixed Costs in US Dollars
Bangladesh	74,455
Colombia	47,038
Uganda	53,976

Fixed costs were primarily incurred in local currency unit (LCU). Our reporting converts from LCU into 2017 US dollars

Table 4 describes the variable costs of the survey reported in USD by (1) survey pricing structure and (2) type of incentive. There was no real pattern found in terms of the combination of pricing mechanisms when using incentives per completed survey. Of note, the costs were overall lower and less heterogeneous when a lottery incentive was applied.

**Table 4:** Distribution of variable costs by type of incentive used and survey pricing mechanism, reported in USD

Country	Incentive per completed survey		Lottery incentive per completed survey	
	Survey pricing per min	Survey pricing via flat fee	Survey pricing per min	Survey pricing via flat fee
Bangladesh	128,668	101,927	99,927	95,787
Colombia^+^	37,244	NA	NA	NA
Uganda^£^	32,073	41,259	32,613	41,259

^+^Variable costs in Colombia do not change based on survey pricing mechanism as only price per minute was conducted

^£^The lottery incentive amount used in Uganda was 20x the individual incentive per completed survey, resulting in the same cost per completed survey across fixed and lottery incentive arms of the trial

Table 5 highlights the distribution of survey-time costs reported in USD across the different scenarios of survey-time pricing and incentive delivery. Survey-time costs are higher when charged a flat fee in Uganda but not in Bangladesh. Bangladesh had a much lower cost when using the lottery incentive mechanism than with a fixed incentive, which is a promising strategy to reduce surveys costs. Uganda does not present similar results because the incentive amount was the same for both lottery and incentive per completed survey.

**Table 5:** Distribution of survey-time costs by type of incentive used and survey pricing mechanism, reported in USD

Country	Incentive per completed survey		Lottery incentive per completed survey	
	Survey pricing per min	Survey pricing via flat fee^#^	Survey pricing per min	Survey pricing via flat fee^#^
Bangladesh	116,387	89,647	93,787	89,647
Colombia^+^	28,080	NA	NA	NA
Uganda	25,444	34,630	25,984	34,630

^+^Variable costs in Colombia do not change based on survey pricing mechanism as only price per minute was made. Incentives based in lottery were not used in Colombia

^#^Survey pricing via flat fee has the same survey-time costs for either type of incentive as the cost incurred for survey time by the payer would only be based on completed surveys

Table 6 reports the costs in USD of offering incentives to those who complete the surveys, by two incentive mechanisms. The first, termed fixed incentive per completed survey, is a smaller incentive amount (equivalent to 1–2 USD in airtime credit) that is given to all participants who complete the survey. The second incentive is delivered through a lottery mechanism with 1 in 20 participants who complete the survey receiving the higher incentive amount. There was a lower incentive cost seen using the lottery mechanism in Bangladesh, and no cost difference was seen in Uganda (this could be because the lottery chance was 1 in 20, and the incentive provided in the lottery was 20 times that of the incentive per completed survey). The patterns across countries was maintained—incentive costs were lowest in Colombia, followed by Uganda, and trailed by Bangladesh which had the highest incentive costs due to the higher number of complete surveys required to obtain a population representative survey under a non-quota scenario.

**Table 6:** Distribution of incentive costs based on type of incentive used and survey pricing mechanism, reported in USD

Country	Incentive per completed survey		Lottery incentive per completed survey	
	Survey pricing per min	Survey pricing via flat fee^#^	Survey pricing per min	Survey pricing via flat fee^#^
Bangladesh	12,280	6140	12,280	6140
Colombia^+^	9164	NA	NA	NA
Uganda^£^	6629	6629	6629	6629

^+^Variable costs in Colombia do not change based on survey pricing mechanism as only price per minute were conducted

^£^The lottery incentive amount used in Uganda was 20x the individual incentive per completed survey, resulting in the same cost per completed survey across fixed and lottery incentive arms of the trial

^#^Survey pricing via flat fee has the same survey-time costs for either type of incentive as the cost incurred for survey time by the payer would only be based on completed surveys

From Tables 4, 5, and 6, it is clear that the main driver of variable costs is survey time. As explained in the sampling section, survey time increases when reaching to subpopulations with lower mobile phone use or ownership (e.g., older adults). Therefore, we assessed and compared the variable costs for a population-level survey nationally representative of all adults versus a similar survey focusing only on adults aged less than 45 years old (Table 7). When comparing both estimates, we found that variable costs reduced between 25 and 58% when surveying adults less than 45 years old compared to a nationally representative sample including all age categories.

**Table 7:** Estimate of variable costs incurred in the collection of nationally representative data for all age groups compared to surveys focusing on persons aged 18–44 years old, reported in US dollars

Country	Variable costs of a nationally representative survey (in USD)^+^	
	For all adults	For persons aged 18–44 years
Bangladesh	128,668	53,629
Colombia	37,244	21,894
Uganda	32,073	23,994

^+^Equivalent changes in costs for this age group are observed in other incentive and survey pricing scenarios, and are available upon request

## Discussion

Mobile phone surveys (MPS) are an emerging method to conduct population health surveys that may provide less expensive and more timely data to monitor NCDs and NCD-related risk factors in LMIC settings. In general, we found that overall costs are proportional to the sample sizes required, and sample sizes are inversely correlated with the mobile phone use; being charged per minute of survey airtime is not always more expensive than being charged a flat fee; using lottery incentive mechanisms to encourage participation is less expensive than a fixed amount for each completion; sampling adult respondents less than 45 years of age requires fewer random digit dials and is much less expensive.

We examined the various cost components of adapting and implementing an MPS in the differing contexts of Bangladesh, Colombia, and Uganda. We found that the total costs of adapting, refining, and deploying one nationally representative survey in these three countries ranged between 47,000 and 75,000 USD. Fixed costs are mostly related to implementation and largely homogenous. Variable costs, which are effectively the sum of survey time (which includes both number of calls and their duration) and the incentive costs, were more heterogeneous than fixed costs. The main variable cost driver is survey time. Total survey costs to deploy a nationally representative MPS is much less expensive than some other comparable household surveys. According to previous reports, the latter might reach costs upwards of up to1 million USD [28]. This is aligned with similar cost comparisons in the area of consumer research [29].

The number of calls required to obtain a nationally representative survey accounts for most of the variable costs across countries. Bangladesh has the highest variable costs, mainly due to the fact that it requires a larger number of calls in order to get the minimum sample size for each age-sex category. This is associated mostly to lower mobile phone use among older adults. Furthermore, it is possible that the literacy levels and the ability to respond to an automated survey like IVR might be more cognitively challenging to the elderly and hence play a role in the lower response rates in this population group [30]. For this reason, we observe that MPS are less expensive to conduct in Colombia and Uganda than in Bangladesh. This is the opposite of what we would see with a face-to-face household survey, where it is expected that labor costs and transport would increase survey costs in those two countries.

We should note that these costing estimates were generated using a non-quota sample. Use of quota sampling methods may reduce costs even further by eliminating survey distribution to individuals whose demographic group has already achieved a target sample. This can be implemented, for example, by imposing quotas by age at the beginning of the survey, so if a quota is already filled that call would only cost 1 min instead of the average 10 min that this survey might take if fully answered.

The limitations of the study are mostly due to the actual implementation of the MPS that served as a basis for cost estimates and in the data that can be collected via MPS. Given that the IVR technical platform used to generate calls and provide incentives that was operative in Uganda and Bangladesh was not operative in Colombia, our study team could not use the same technology provider in all study three countries. Nonetheless, the platforms were generally similar and the agreements reached with both platforms (one covering Bangladesh and Uganda, and the other one covering Colombia) were very similar. This costing study was conducted as part of the formative phase of the project and therefore costs are based on a deployment that is managed by collaborating academic/research institutions, and not national or international organizations. A full-scale national implementation may have different components that we may not have accounted for, including the possibility of further savings due to scale or additional unexpected fixed costs.

We evaluated costs for an IVR MPS on its own; however, MPS does not necessarily need to be implemented to the exclusion of other household or site-based survey methods. It may be used in conjunction with such modalities to potentially increase efficiency of traditional methods for monitoring risk factors. Different phone-based delivery modalities can also be mixed, for example, IVR can be used for younger populations and computer-assisted telephone interviewing (CATI) or call center may be better options for surveying older populations, as these strategies are more interactive and the respondent has a chance to get clarification from the human interviewer, which is not possible in IVR [18].

Mobile phone surveys can be included as part of the efforts to reduce costs and improve responsiveness in monitoring and evaluation of NCD risk factors. This study fills a gap in knowledge about costs of deployment within three regionally and socio-demographically different countries to help decision-makers to take better informed decisions related to the potential use of these surveys to evaluate NCD policy impact, or to monitor NCD risk factors more generally, within fiscal constraints. This study therefore provides data for better understanding of the economic feasibility of conducting MPS for risk factors on NCDs in LMICs.

## Conclusion

The broader patterns in survey costs become evident. Mobile phone costs through IVR are relatively inexpensive; being charged per minute of survey airtime costs is not always more expensive than being charged a flat fee; using lottery incentive mechanisms to encourage participation is generally less expensive than a fixed amount for each completion; sampling adult respondents less than 45 years of age requires fewer random digit dials and number of phone calls deployed and is much less expensive.

## Supplementary Information

**Additional file 1.** Appendix 1

## Data Availability

The datasets used and/or analyzed during the current study are available from the corresponding author on reasonable request.

## Abbreviations

CATI: Computer-assisted telephone interviewing
CHEERS: Consolidated Health Economic Evaluation Reporting Standards
D4H: Bloomberg Philanthropies Data for Health Initiative
IVR: Interactive voice response
LCU: Local currency units
LMIC: Low- and middle-income countries
MPS: Mobile phone surveys
NCD: Non-communicable diseases
RDD: Random digit dialing
ROI: Return on investment
STEPs: Stepwise Approach to Surveillance
USD: United States dollars
