# Supplementary material for: A cost study for mobile phone health surveys using interactive voice response for assessing risk factors of noncommunicable diseases
Source: Popul Health Metr. 2021 Jun 28;19:32. doi: 10.1186/s12963-021-00258-z (PMC8240284; doi:10.1186/s12963-021-00258-z)
Supplement: Supplementary file 1 — Additional file 1. Appendix 1 [file 12963_2021_258_MOESM1_ESM.docx]

**Appendix 1**

Age-sex distribution by country for complete calls and calls in which respondent answered the age question (both complete and incomplete) with fixed incentive

Bangladesh age-sex distribution for those who complete survey and overall

|  | Only complete surveys | | All who answered age question | |
| --- | --- | --- | --- | --- |
| Age group | Male | Female | Male | Female |
| 18-29 | 236 | 31 | 405 | 40 |
| 30-44 | 64 | 11 | 131 | 18 |
| 45-59 | 15 | 3 | 28 | 3 |
| 60+ | 6 | 1 | 22 | 4 |

Colombia age-sex distribution for those who complete survey and overall

|  | Only complete surveys | | All who answered age question | |
| --- | --- | --- | --- | --- |
| Age group | Male | Female | Male | Female |
| 18-29 | 90 | 85 | 113 | 113 |
| 30-44 | 56 | 71 | 67 | 91 |
| 45-59 | 29 | 36 | 37 | 46 |
| 60+ | 14 | 10 | 18 | 15 |

Uganda age-sex distribution for those who complete survey and overall

|  | Only complete surveys | | All who answered age question | |
| --- | --- | --- | --- | --- |
| Age group | Male | Female | Male | Female |
| 18-29 | 233 | 72 | 338 | 116 |
| 30-44 | 61 | 11 | 86 | 24 |
| 45-59 | 9 | 1 | 12 | 1 |
| 60+ | 2 | 0 | 3 | 1 |
